# An overview of probiotic camel milk as a nutritional beverage: Challenges and perspectives

**DOI:** 10.1002/fsn3.4298

**Published:** 2024-06-24

**Authors:** Fereshteh Ansari, Hadi Pourjafar, Shohreh Alian Samakkhah, Esmaeel Mirzakhani

**Affiliations:** ^1^ Razi Vaccine and Serum Research Institute, Agricultural Research, Education and Extension Organization (AREEO) Tehran Iran; ^2^ Research Center for Evidence‐Based Medicine Health Management and Safety Promotion Research Institute, Tabriz University of Medical Sciences Tabriz Iran; ^3^ Iranian EBM Centre: A Joanna Briggs Institute Affiliated Group Tabriz Iran; ^4^ Dietary Supplements and Probiotic Research Center Alborz University of Medical Sciences Karaj Iran; ^5^ Department of Food Hygiene, Faculty of Veterinary Medicine Amol University of Special Modern Technologies Amol Iran; ^6^ Department of Food Science and Technology, Faculty of Nutrition and Food Sciences Tabriz University of Medical Sciences Tabriz Iran

**Keywords:** camel milk, dairy beverage, dairy food, functional food, postbiotic, probiotic

## Abstract

There are challenges such as standardization for commercialization and guaranteeing sensory characteristics in camel milk processing. This review gathers a general view of the probiotic camel milk, its contents, its health aspects, and its industrial production. One of the potential candidates of a healthy food product is “probiotic camel milk” which contains several nutritional elements including Lactic acid bacteria and Bifidobacteria and postbiotics such as endopolysaccharides, exopolysaccharides, numerous beneficial enzymes, short‐chain fatty acids, teichoic acids, peptides, peptidoglycan‐derived neuropeptides, cell surface proteins, different vitamins, plasmalogens, and different kinds of organic acids. It should also be considered that camel milk generally has some advantages over cow milk like its health‐beneficial antidiabetic, hypoallergenic, and anticancer properties. As a result, it is gaining much attention from both consumers and manufacturers, and the global probiotic market trend is growing. Although there are obstacles in standardizing processing techniques and maintaining sensory excellence, the health benefits, economic prospects, and adaptability of camel milk and its probiotic variations create a promising avenue for continued research and advancement. Therefore, developing standardized processing techniques and sensory evaluation methods for probiotic camel milk can unlock its full potential as a nutritious beverage, offering a promising solution for consumers seeking healthy and functional food products.

## Graphical abstract

Camel milk has shown many advantages over milk from other animals and presents promising opportunities for health and economic sectors. Probiotic isolates from camel milk, such as LAB and Bifidobacteria, show potential as probiotics with beneficial properties. The predominance of β‐casein and the deficiency of β‐lactoglobulin, along with high nutritional value, make camel milk consumption suitable for humans, especially children who are sensitive to cow's milk. Additionally, postbiotics derived from camel milk exhibit antioxidant, antidiabetic, antiproliferative, and antihypertensive effects.

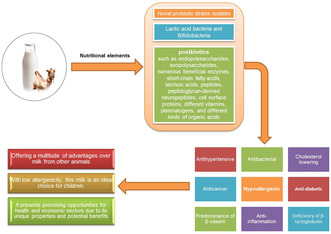

## 1 INTRODUCTION

Today, with the increase in food standards and consumers becoming aware of the impact of foods on human health and their ability to prevent diseases, the views and interests of people in the community regarding healthy and beneficial foods have changed (Granato et al., 2010). Probiotics are an example of food supplements that are administered in the form of live bacteria, and if consumed in sufficient quantities, in addition to nutritional properties, they will bring health benefits to the host (Perricone et al., 2015). Probiotics are nothing new and have been in their food ever since humans started consuming fermented foods (Vijaya Kumar et al., 2015). Lactic acid bacteria (LAB), Bifidobacterium, and Lactobacillus are the most common probiotic genera that have been used. These genera have been recognized as the generally recognized as safe (GRAS), so their consumption does not endanger human health (Penner et al., 2005; Ranadheera et al., 2010; Salminen et al., 1998). LAB can exert their effects in one or several different ways. For example, they can restrict the physiological environment of pathogenic microorganisms through the reduction of pH due to the breakdown of complex carbohydrates and as a result, releasing organic acids such as short‐chain fatty acids (SCFAs) and lactate (LeBlanc et al., 2017), or through the creation of antibiotic‐like compounds such as bacteriocin‐like substances (Cholakov et al., 2021). LAB are known to be the agent that confers several nutritional, health, and therapeutic properties to fermented milk products (Zhu et al., 2011).

**FIGURE 1:**
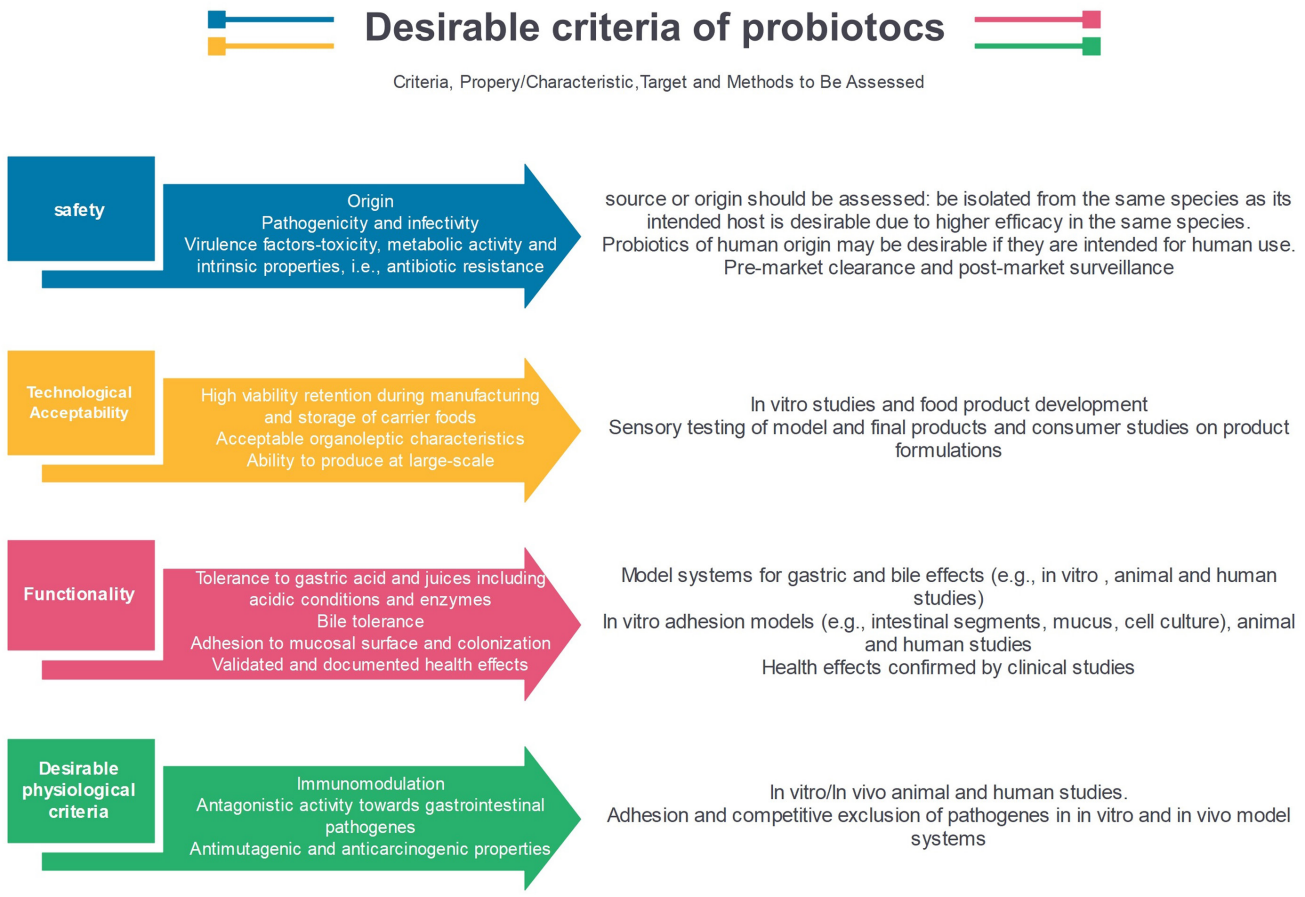
Desirable criteria for the selection of probiotics in food applications.

Several shreds of evidence show that probiotics are involved in the prevention and treatment of urogenital tract diseases (Grin et al., 2013), respiratory diseases (Mortaz et al., 2013), and gastrointestinal (GI) diseases (Milner et al., 2021). Probiotics can exert their beneficial effects mainly by helping to maintain gut homeostasis and preventing pathogens (D'Aimmo et al., 2007). Immunomodulatory, antioxidant, and anti‐carcinogenic activity (Brasiel et al., 2020), reduction of lactose intolerance (Oak & Jha, 2019), reduction of hypertension (Khalesi et al., 2014), reduction of cholesterol levels, and reduce the risk of cardiovascular disease (Sivamaruthi et al., 2021), improving nutrient bioavailability, and increasing the nutritional value of foods (Hariri et al., 2018) are among the benefits of probiotics for health. Therefore, probiotics can be used to prevent and treat various diseases. The origin of these health benefits of probiotics may be their function in the intestine, or their growth and metabolism during the production of fermented foods, which will be effective after entering the host's body (Lourens‐Hattingh & Viljoen, 2001; Rašić, 2003). Although fermented dairy products have been on the food market for more than a decade, the dairy industry continues to expand through the production of probiotic products (Saad et al., 2013; Tripathi & Giri, 2014). On the other hand, to choose probiotic bacteria, different parameters should be considered (Figure 1).

Probiotic survival and its potential effects during gastric transit depend on the physicochemical characteristics of the food used as a carrier for probiotic delivery, such as pH and buffering capacity. Food formulation with an appropriate pH range and high buffering capacity increases the pH of the digestive tract and thus increases the survival of probiotics (Kailasapathy & Chin, 2000). One of the products that have been introduced as a suitable carrier for delivering probiotics to the GI tract is dairy products (Ross et al., 2002). Recent advances in research on probiotics promise the possibility of producing new food products based on dairy and milk. In the present study, we will review the current perspectives and challenges regarding camel milk as a nutritious drink. The review aims to highlight the nutritional and therapeutic benefits of camel milk, investigate the probiotic isolates of camel milk and its potential as a source of probiotics, and promote its use for human consumption, as well as the economic benefits of camel milk production.

## 2 CAMEL MILK AND ITS NEW APPROACHES

The camel belongs to the Camelidae family, which includes two genera: Lama and Camelus. Genus Camelus also includes camels with one hump (Dromedary camels or Camelus dromedarius) and camels with two humps (Bactrian camels or Camelus bactrianus) (Patel, 2018). Traditionally, the camel was usually used as a pack animal in agriculture and transportation, but the use of this animal for this purpose has been greatly reduced after the mechanization of transportation and agricultural operations (Kumar, Verma et al., 2016). Today, camels have become important due to their special properties such as immunogenic compounds in milk (Burren et al., 2014). While the risk of desertification has increased due to the over‐exploitation of water and land resources (Heshmati & Squires, 2013), it has been reported that camels can produce more milk and have a longer lactation period than other species in the harsh conditions of the desert ecosystem, while their feed requirements are modest (Farah et al., 2007). Its average milk production is between 3 and 10 L per day during the lactation period (Farah et al., 2007). Depending on factors such as water access, proper nutrition, and livestock care, milk production can be increased by up to 20 L per day (Kumar, Verma et al., 2016). Dairy consumption has problems and is associated with some health risks, such as lactose intolerance, high cholesterol, and fat content, and milk protein allergies (Prado et al., 2008; Vijaya Kumar et al., 2015). The comparison between cow milk and camel's milk shows that solids, percentage of fat, and total protein are more in cow milk, and on the contrary, protective proteins and percentage of total salts are more in camel's milk (Benkerroum, 2008; Konuspayeva et al., 2007; Singh et al., 2006; Wang et al., 2011). The percentage of ingredients in camel milk is on average 13.0% total solids, 3.4% protein, 3.5% fat, 4.4% lactose, and 0.79% ash (Abdullahi, 2019). This amount of lactose is easily metabolized in the body of subjects with lactose intolerance (Al‐Juboori et al., 2013; Cardoso et al., 2010; Konuspayeva et al., 2019). Fe, Na, Cu, and K among the salts that were found in camel milk more than in bovine milk (Muthukumaran et al., 2022). The amount of cholesterol in camel milk is 30 mg per 100 g of dry matter, which is very low. About 96% of the fat component of this milk is triglycerides (Salwa & Lina, 2010). The size of the fat globule in camel milk is smaller compared to the milk of animals such as bovine, buffalo, and goat, and its higher digestibility is also attributed to this feature (Meena et al., 2014). Another important component that affects the bioactive properties and nutritional value of milk is proteins. About 50–87% of the proteins in camel milk are made up of caseins, which is about 80% in cow's milk (Maqsood et al., 2019). In terms of molecular weight, camel milk proteins have a higher weight compared to their counterparts in bovine milk (Khalesi et al., 2017). In addition, the comparison between casein micelles shows that the size of the micelles in camel milk is about twice as big as the micelles of cow's milk, which has caused camel milk to be technologically and functionally different (Ibrahim & Khalifa, 2015; Zhao et al., 2015). For a long time in different parts of the world, the consumption of camel milk by humans has been recognized as a suitable alternative to cow's milk (Stahl et al., 2006). Also, according to microbiological evaluations, it has been determined that camel milk meets the existing international standards for cow milk. The standard plate count of camel milk is reported to be lower than that of cow milk (Abuelnaga et al., 2022). Camel milk inhibits both gram‐negative and gram‐positive bacteria, including *Salmonella typhimurium*, *Escherichia coli*, *Staphylococcus aureus*, and *Listeria monocytogenes* (El Sayed et al., 1992; Kumar et al., 2016a, 2016b). Camel milk whey proteins such as lactoferrin (LF), immunoglobulins, serum albumin, and peptidoglycan recognition protein (PGRP) have shown antibacterial, antiviral, and immunological properties (Farah, 1993; Felfoul et al., 2017; Kappeler et al., 2004; Merin et al., 2001).

Recently, it has been suggested that children who are allergic to cow milk use camel milk instead. The low allergenicity of mother milk is attributed to the low percentage of α‐CN, high percentage of β‐CN (El‐Agamy et al., 2009), deficiency of β‐lactoglobulin (Kappeler et al., 1998), and similarity of the immunoglobulins (Shabo et al., 2005). Since camel milk proteins and their percentage are almost similar to human milk proteins (El‐Agamy et al., 2009), and also due to the predominance of β‐casein over other fractions of casein (αS1‐casein, αS2‐casein, and κ‐casein) in camel milk (Mohamed et al., 2020), which is an important factor in creating biological properties such as antiallergic and more digestible properties, it is expected to cause little allergenicity (Abou‐Soliman, 2005; El‐Agamy, 2009). Also, camel milk has a beneficial effect as a supplement to mother milk (Davies & Law, 1980). Camel milk is known for its wonderful healing properties (Table 1). Camel milk can show therapeutic effects in a wide range of diseases. Cancer (Krishnankutty et al., 2018), diabetes (Mirmiran et al., 2017), flatulence (Cardoso et al., 2010), allergy (Ehlayel et al., 2011), tuberculosis (Mal et al., 2006), and liver cirrhosis (Sadek et al., 2016) are among these diseases. Recently, camel milk has been used to help treat liver damage caused by alcohol consumption, lactose intolerance, infant diarrhea, autism, and insulin‐dependent diabetes mellitus (IDDM) (Patel et al., 2022). There are amounts of insulin‐like molecules in camel milk (Agrawal et al., 2005). In this regard, it has been stated that camel milk is effective in the function of residual B‐cell in type 1 diabetes (Agrawal, Budania et al., 2007; Agrawal, Saran et al., 2007). According to the evidence, the prevalence of diabetes is low in communities where camel milk is used (Agrawal, Budania et al., 2007). Many of these therapeutic properties that camel milk has are often attributed to its composition, especially the proteins, peptides, and fatty acids present in it (Alavi et al., 2017; Khalesi et al., 2017; Maqsood et al., 2019). Compared to the milk of another species, camel milk has a stronger protective system and it has been claimed that can be kept at 37°C for about 8 h and at refrigerator temperature for more than 7 days (Muthukumaran et al., 2022). Several bioactive components in milk cause these potential health benefits (Kumar et al., 2016a, 2016c). The camel population worldwide has increased from about 13 million in 1961 to more than 35 million by 2018 (Faye, 2020). These data show that the awareness of the properties of camel milk and its use as a health‐promoting product is gradually increasing, which can be one of the reasons for the increase in the camel population.

**TABLE 1:** Therapeutic properties of camel milk and its ingredients.

Disease under treatment	Camel milk/ ingredients	Animal model/cell line/assay used	Mechanism	References
Diabetes	Whey protein	Human liver cancer cell line (Hep‐G2) and human embryonic kidney cell line (HEK‐293)	Stimulating the insulin receptor and increasing glucose absorption	Ashraf et al. (2021)
	Protein hydrolysates	Streptozotocin (STZ)‐induced diabetic rats	Reduction of blood glucose levels and powerful hypoglycemic effect	Kilari et al. (2021)
	Whey Protein hydrolysates	Using the colorimetric method	Inhibition of ∝‐glucosidase and ∝‐amylase	Baba et al. (2021)
Hypertension	Casein hydrolysates	Using the colorimetric method	Inhibition of ACE^a^	Rahimi et al. (2016)
	Protein Hydrolysates	Using the colorimetric method	Inhibition of ACE and anti‐inflammatory responses	Mudgil et al. (2019)
	Protein and lipid fractions	Using the colorimetric method	Inhibition of ACE	Maqsood et al. (2019)
Cancer	Milk	Breast Cancer Cell Line (MCF‐7) and Human Colorectal Cancer Cell line (HCT‐116)	Induction of autophagic cell death	Krishnankutty et al. (2018)
	Milk	Human Breast Cancer Cell Line (MCF‐7) and Human Hepatoma Cell Line (Hep‐G2)	Regulation of apoptosis	Korashy, Maayah et al. (2012)
	Milk	Murine Hepatoma Hepa 1c1c7 Cell Line	Modulation of cancer‐related genes expression (Gsta1, Nqo1 and Cyp1a1)	Korashy, El Gendy et al. (2012)

^a^ ACE, angiotensin‐1 converting enzyme.

## 3 PROBIOTIC ISOLATES OF CAMEL MILK

Some investigations have revealed that camel milk has a number of advantages over cow milk, including being rich in some rare minerals such as molybdenum, as well as having high levels of natural antimicrobial compounds (Baghiani et al., 2003; Elagamy, 2000; Elagamy et al., 1996; Rahmeh et al., 2019; Yassin et al., 2015). Therefore, researchers are more interested in working on camel milk, including the separation and identification of some possible probiotics. Bifidobacteria and LAB seem prime candidates to be probiotics (Prasad et al., 1998; Reid, 1999). However, bacteria isolated from this or other genera must have some probiotic properties, such as being safe and having beneficial effects on the host body (GRAS) (Bubnov et al., 2018). Some other principles have been applied to consider novel probiotic strain isolates including tolerance to harsh environments of the GI lumen (gastric acid, intestinal enzymes, and bile salts), tolerance to adverse conditions in fermented foods, lysis of bile salts, antimicrobial properties, ability to lower cholesterol, and being non‐hemolytic (Campana et al., 2017; Cho et al., 2020; Gharib, 2020; Kumar et al., 2015). Some research suggests that camel milk can be a major source of isolation for various new probiotic strains, including LAB (Nagyzbekkyzy et al., 2020; Rezaei et al., 2020; Sharma et al., 2021; Zhao et al., 2020). However, the number of studies related to the isolation and identification of possible probiotics in camel milk is small, due to increased camel milk production in countries such as Saudi Arabia, UAE, Somalia, and other similar Arab countries and Iran has recently paid more attention to this issue (Al Kanhal, 2010; Fguiri et al., 2016; Mahmoudi et al., 2016; Yam et al., 2014).

Typically, a number of bacterial cultures are employed for the initial isolation of potential probiotic bacteria, the most significant of which is de Man Rogosa Sharpe (MRS) broth/agar medium (Shah, 2000). For specific isolation of certain genera and species of probiotics, elective/selective culture media are usually used in which the basic culture medium is the same as MRS, and then about 1% of active factors such as different types of carbohydrates (starch, mannitol, sorbitol, Glucose, fructose, maltose, etc.) or antibiotic agents (such as vancomycin, etc.) and other specific factors for isolation of each genus and species are added to the base culture medium (MRS agar) (Khedid et al., 2009; Shah, 2000). For example, MRS‐Salicin agar for the probiotic of *L*. *acidophilus* LA5 (Abdolhosseinzadeh et al., 2018; Mirzaei et al., 2012), and MRS‐Glucose‐vancomycin agar for *L*. *rhamnosus* GG (Ansari et al., 2017; Pourjafar et al., 2018, 2020). Moreover, several culture media for selective/elective enumeration of *Bifidobacterium* spp. and *Lactobacilli* and *Bifidobacterium* spp. have been previously recommended in dairy products such as MRS‐maltose agar, MRS‐bile agar, MRS‐glucose‐vancomycin agar, MRS‐sorbitol agar, MRS‐IM agar, M17 agar, MBG agar, RCPB agar, TPPY‐E agar, AMC agar, DP agar, MRS‐LP agar, TOS‐NNLP agar, BIM‐25 agar, BL‐OG agar, and so on (Akalın et al., 2018; Davis, 2014; Homayouni et al., 2018; Karimi et al., 2012; Pourjafar et al., 2016; Shah, 2000; Vinderola et al., 2019). According to the findings of previous work, MRS agar having the structure of elective/selective and various factors is entirely considered appropriate when enumerating probiotics in dairy products (Akalın et al., 2018; Daniela et al., 2011; Van de Casteele et al., 2006; Vinderola et al., 2019).

In fact, the ability to ferment certain carbohydrates with or without gas production, the ability to resist certain antibiotics, the ability to use certain protein compounds, and other similar items are unique features of each probiotic. By designing specific culture media according to the specific characteristics of each probiotic, in the initial stage, different probiotics in each food can be isolated and differentiated to a large extent at least in terms of the genus. In the next steps, using advanced genetic methods and considering the physiological characteristics of the isolates, allow for the exact identification of probiotics. There have been a few publications in the literature in relation to the isolation and identification of probiotics from camel milk. In some of these studies, isolated microorganisms, especially from the LAB family, lacked probiotic properties, and in some studies, new strains were identified that have probiotic properties. The study carried out by Fguiri et al. (2016) lacks probiotic properties such as acid and bile tolerant capabilities, hemolytic pattern, antimicrobial ability, cholesterol elimination ability, and employment of conventional non‐DNA‐based techniques for the identification of bacterial isolates. Also, some other studies including the investigations of Soleymanzadeh et al. (2016), and Yateem (2008) lack several probiotic parameters to offer any relevant evidence that the separated LAB carries probiotics properties. In fact, they tried to recognize the isolated LAB without employing evidence obtained from the identification of isolates via DNA‐based technique. Findings of recent studies have shown that various possible probiotic bacteria can be isolated from camel milk and have examined probiotic properties, for instance, physiological characteristics, cholesterol‐eliminating characteristics, acid and bile tolerance capabilities, bile salt degradation, antimicrobial and hemolytic activities, cell superficial characteristics (such as auto‐aggregation, hydrophobicity, and co‐aggregation), exopolysaccharides (EPS) creation potential, growth ability in fermented foods, resistance toward lysozyme and antibiotics, and 16S rRNA sequencing or 16S rDNA sequencing to identify possible potential probiotic isolates (Abushelaibi et al., 2017; Ayyash, Abushelaibi et al., 2018; Ayyash et al., 2020; Sharma et al., 2021).

Abushelaibi et al. (2017) studied the properties of possible probiotic LAB isolated from camel milk. In their investigation, physiological characteristics, cell surface properties acid and bile tolerant potential, bile salt hydrolysis, EPS creation, cholesterol elimination, antimicrobial and hemolytic activities, resistance to six antibiotics and lysozyme, and fermentation profile were surveyed. 16S rRNA sequencing method was employed to recognize six probable LAB isolates. Overall, entirely identified LAB (*L*. *lactis* KX881782, *L*. *lactis* KX881768, *L*. *plantarum* KX881779, and *L*. *plantarum* KX881772) displayed auto‐aggregation ability, high co‐aggregation, high cholesterol elimination capacity, robust antimicrobial action, and EPS creation. Also, *L*. *lactis* KX881782, *L*. *lactis* KX881768, *L*. *plantarum* KX881779, and *L*. *plantarum* KX881772 showed significant cholesterol elimination abilities. Likewise, *L*. *lactis* KX881782 and *L*. *plantarum* KX881779 presented actual promising fermentation profiles (Abushelaibi et al., 2017).

Ayyash, Abushelaibi et al. (2018) investigated the isolation of LAB, namely *Enterococcus* and *Streptococcus* from camel milk and explored their probiotic features. Similar to the previous study (Abushelaibi et al.), all required possible probiotic properties were investigated. 16S rDNA sequencing was employed to recognize the isolates and to obtain GenBank accession numbers. LAB isolates displayed cholesterol‐dropping and pathogens prevention characteristics. Auto‐aggregation and hydrophobicity consequences discovered robust attachment abilities of the separated LAB. Recognized LAB showed a promising fermentation profile. Also, the resistance of LAB isolates to lysozyme and to 60°C was high. The results of this study disclose that the LAB isolates particularly *S*. *equinus* KX881778 and *E*. *faecium* KX881783 can be admirable probiotic strains (Ayyash, Abushelaibi et al., 2018).

In the same vein, Sharma et al. (2021) examined the identification and probiotic potential of LAB from camel milk. Chosen LAB were recognized as *L*. *lactis*, *L*. *plantarum*, and *Enterococcus lactis*, and their potential was verified via antimicrobial activity, tolerance and de‐conjugation of bile salts, superficial hydrophobicity, as well as adhesion ability. Selected LAB exhibited antimicrobial properties in contradiction of an extensive variety of pathogenic bacteria such as *E*. *coli*, *Pseudomonas aeruginosa*, *Staphylococcus aureus*, and *Bacillus cereus*. Adhesion surveys proved robust adhesion ability with high hydrophobicity (Sharma et al., 2021).

In general, camel raw milk and some of the fermented products produced from it can be a source of various probiotic strains. According to several studies, examples of which have already been mentioned. Different types of bacterial species such as *L*. *fermentum*, *L*. *lactis*, *L*. *plantarum*, *E*. *faecium*, *L*. *casei*, and *E*. *lactis* have been found and isolated as the primary probiotics in camel milk, the same probiotic bacteria, can be used to produce other probiotic products. Table 2 displays some selected studies of the identification of possible probiotic strains from camel milk. However, the number of available studies on the identification and isolation of potential probiotic bacteria from camel milk is small; therefore, extensive studies are needed to identify new probiotic strains.

**TABLE 2:** Some selected studies of the identification of possible probiotic strains from camel milk.

Sample	Possible probiotic strains	Probiotic characteristics surveyed	Identification methods	References
Raw camel milk	*L*. *lactis* KX881768, *L*. *plantarum* KX881772, *L*. *lactis* KX881782, *L*. *plantarum* KX881779	Physiological and cell surface characteristics (hydrophobicity, auto‐aggregation, co‐aggregation), eliminating of cholesterol, bile and acid tolerance, lysis of bile salt, exopolysaccharides (EPS) creation, hemolytic activity, antimicrobial activity, fermentation profile (growth, pH, and proteolysis) resistance against lysozyme, and six antibiotics	PCR: 16S rRNA sequencing	Abushelaibi et al. (2017)
Raw camel milk	*Enterococcus faecium* KX881783, *Streptococcus equinus* KX881778	Physiological and cell surface characteristics (hydrophobicity, co‐aggregation, auto‐aggregation), eliminating of cholesterol, bile and acid tolerance, lysis of bile salt, EPS creation, hemolytic activity, antimicrobial activity, fermentation profile (growth, pH, and proteolysis) resistance against lysozyme, and six antibiotics	PCR: rDNA sequencing	Ayyash, Abushelaibi et al. (2018)
Raw camel milk	*L*. *plantarum* C70 (accession number KX881779)	EPS production	–	Ayyash et al. (2020)
Raw camel milk	*L*. *lactis*, *Enterococcus lactis*, *L*. *plantarum*	Antimicrobial activity, tolerance and de‐conjugation of bile salts, curd formation, surface hydrophobicity, adhesion property	PCR: 16S rRNA sequencing	Sharma et al. (2021)
Raw Moroccan camel milk	*Weissella confusa*, *Weissella cibaria*, *Enterococcus durans*	Acidifying ability, proteolysis, autolysis, lipolytic activities, diacetyl and exopolysaccharides production, tolerance to gastrointestinal (GI) conditions. The auto‐aggregation, hydrophobicity, and antioxidant activity, antibacterial activity, antibiotic resistance, hemolytic or DNase activities	PCR: 16S rDNA sequencing	Mercha et al. (2020)
Xinjiang camel milk yogurt	*L*. *rhamnosus* GG (ATCC53103), *L*. *paracasei* FM‐LP‐4	2, 2‐diphenyl‐1‐picrylhydrazyl scavenging activity, adhesion potential, stress tolerance characteristics (bile, acidity, and osmotic pressure), antioxidant property (in vivo), prevented the biosynthesis of malondialdehyde (MDA) and inhibited protein carbonyl in a dose‐dependent way, and enhanced superoxide dismutase (SOD) and glutathione peroxidase (GSH‐Px) activities	PCR: 16S rDNA nucleotide sequencing	Wang et al. (2016)
Raw milk of Iranian one humped camel	*Pediococcus pentosaceus*, *Enterococcus faecium* Y‐2, *E*. *faecium* JZ1‐1, *E*. *faecium* E6, *E*. *durans*, *E*. *lactis*, *Leuconostoc mesenteroides*, *Lactobacillus casei*, *Weissella cibaria*	Antimicrobial activity, acid and bile tolerance	PCR: 16S rDNA and Internal Transcribed Spacer (ITS) region between the23S rRNA and 16S genes, then separated and clustered via the Amplified Ribosomal DNA Restriction Analysis (ARDRA) technique	Davati et al. (2015)
Raw camel milk	*Bifidobacterium longum* B‐11	Survival at high bile salt and low pH conditions, cholesterol assimilation, antioxidant potential, adhesion potential, resistant to fusidic acid, neomycin, nalidixic acid, polymyxin B, gentamicin, rifampicin, kanamycin, and streptomycin, production of EPS, antagonistic properties, decrease of nitrite	PCR: 16S rRNA gene sequencing	Yasmin et al. (2020)
Iranian raw camel milk	*Enterococcus faecium* MN994352, *Lactococcus lactis* MN994342, *Lactococcus lactis* MT032418, *Leuconostoc mesenteroides* MT032416, *Leuconostoc mesenteroides* MN994377, *Leuconostoc mesenteroides* MN994378, *Leuconostoc mesenteroides* MT032415	Survival in gastric fluid, antioxidants, lipolytic, and proteolytic activity, antibacterial activity, production of EPS, ability in co‐aggregation with pathogens	PCR: 16S rRNA gene sequencing	Rezaei et al. (2020)
Mongolian camel milk products	*L*. *paracasei* subsp. *paracasei*	Hepatoprotective activity (liver damage inhibitor in the interference of inflammation‐based liver illness), fermentation of 49 different carbohydrates, simulated gastric fluid tolerance, bile tolerance, ability to adhere Caco‐2 cells, fermentation profiles	PCR: 16S rDNA sequencing	Xu et al. (2019)
Raw camel milk, traditional fermented camel milk (Chal)	*Lactococcus lactis* KMCM3, *Lactobacillus helveticus* KMCH1	Antibiotic resistance ability, hemolysis ability, adhesion aptitude to hydrocarbon, auto‐aggregation and co‐aggregation rates, resistance to low pH and high bile salts, survival under GI circumstances, antibacterial potential	PCR: 16S rDNA gene (1500 bp) sequencing	M. Mahmoudi et al. (2019)
Raw camel milk	*Lactobacillus paracasei* ssp. *paracasei*, *Lactobacillus plantarum*, *Lactobacillus rhamnosus*, *Lactobacillus fermentum*, *Lactobacillus brevis*	Temperatures, NaCl and pH concentrations impact on growth, antibiotic resistance, survival in GI situations, bacteriocin‐like activity, fermentation profiles	Production of acids using carbohydrates and related compounds via API 50 CH kits and CHL media. These results were joined to the apiweb™ identification software with database (V5.1), applying the phenotypic data to predict a species identity	Muna and Adel (2014)
Raw camel milk	*Pediococcus pentosaceus* CM16, *Lactobacillus brevis* CM22	Antimicrobial activity, characterization of bacteriocins produced, Anti‐Listeria activity of the bacteriocins	PCR: 16S rRNA sequencing	Rahmeh et al. (2019)
Raw camel milk	*Enterococcus faecium* LCW 44	Antibacterial activity, resistance to GI stresses, prevention of *L*. *monocytogenes* in medium simulating colonic nutrients, adhesion and competition assess on HT‐29 and Caco‐2 cells	PCR: 16S rDNA sequencing	Vimont et al. (2017)
Raw camel milk	*Leuconostoc mesenteroides*, *Lactobacillus plantarum*, *Weissella paramesenteroides*, *Weissella confuse*	Antagonistic activity in contradiction of *Staphylococcus aureus* subsp. *aureus* PTCC 1431 and *E*. *coli* ATCC 25922 (in vitro), proteolytic activity	PCR: 16S rRNA sequencing	Edalati et al. (2019)
Traditional butter made from camel milk	*Lactobacillus plantarum* SH5, *L*. *plantarum* SH12, *L*. *plantarum* SH24, *L*. *plantarum* SH32	Bile and acidic pH tolerance, antibacterial activity, antibiotic resistance ability, acidification activity, proteolytic activity, survival percentage after freeze‐drying, polysaccharides or hemolysin production ability	–	Maurad and Meriem (2008)
Algerian raw camel milk	*Leuconostoc mesenteroides* subspecies *mesenteroides* B7, *Leuconostoc mesenteroides* subspecies *mesenteroides* Z8	Bile, acidic pH, and pepsin tolerance, antimicrobial activity, bacteriocin production ability, identification of the proteinaceous nature of the inhibitory factor, hemolytic activity, antibiotic sensitivity test	PCR: 16S rRNA sequencing	Benmechernene et al. (2013)
Morocco one humped camel milk	*Lactococcus lactis* subsp. *lactis*, *L*. *helveticus*, *L*. *casei* subsp. *casei*, *L*. *plantarum Streptococcus salivarius subsp*. *thermophilus*	Phenotypic characterization: shape of bacteria, acetoin production ability, and NaCl tolerance, carbohydrate fermentation profile: the fermentation capability of starch, melezitose, arabinose, galactose, cellobiose, mannitol, amygdalin, lactose, trehalose, salicin, fructose, glucose, maltose, mannose, melibiose, rhamnose, sucrose, ribose, sorbitol, raffinose, and xylose in MRS broth, gas production ability	–	Khedid et al. (2009)
Tunisian camel raw milk	*L*. *fermentum* spp., *L*. *plantarum* spp.	Bile salts, pH 2, pepsin, and pancreatin tolerance mucin degradation, hemolytic activity, antibacterial activity, antibiotic resistance ability, adhesion to human Caco‐2 and HT29‐MTX epithelial cells	PCR: 16S rRNA sequencing	Mahmoudi et al. (2016)

## 4 POSTBIOTICS

The term postbiotics is also recognized as cell‐free supernatants (CFS) or metabolites; soluble substances secreted by active microorganisms through their lifecycle or emitted later bacterial lysis.

Postbiotics have several interesting physical functions such as antioxidant, anti‐inflammatory, antihypertensive, immunomodulatory, hypercholesterolemia, anti‐obesogenic, and antiproliferative effects (Aguilar‐Toalá et al., 2018). These categories make the performance of postbiotics better understood and used as a guide for their industrial and clinical application.

Camel milk is regarded as a good source of beneficial microbes, especially LAB and Bifidobacteria. LAB have been shown to be isolated from raw and fermented camel milk products in several studies, and there are documents of successful isolation of Bifidobacteria from camel milk (Yasmin et al., 2020). The presence of postbiotics in camel milk has also been scrutinized.

The water‐soluble extract of camel milk containing probiotics (*L*. *reuteri‐*KX881777, *L*. *plantarum*‐KX881779, *L*. *plantarum*‐KX881772, and strain *L*. *plantarum* DSM2468) has been compared with bovine milk with the same strains of probiotics. The anticancer and antioxidant potential of bovine milk is lesser than fermented camel milk. In this study α‐amylase and α‐glucosidase inhibition, angiotensin‐converting‐enzyme (ACE)‐inhibition, and inhibition of production of MCF‐7, Caco‐2, and HELA cells after treatment with water‐soluble extract of fermented milk have been measured (Ayyash, Al‐Nuaimi et al., 2018). In a similar study, water‐soluble extract of camel milk and bovine milk was examined after administration of *L*. *lactis* KX881782 (L. K782) (the indigenous probiotic of camel milk) and *L*. *acidophilus* DSM9126 (L. DSM) (nonindigenous strain). The results revealed that not only probiotic camel milk has markedly higher health‐beneficial effects than bovine milk but also the indigenous probiotic of camel milk shows a better function as a starter than the other strains. Antidiabetic activity via α‐glucosidase and α‐amylase inhibitions, antihypertensive activity, antiproliferative activity via angiotensin‐converting enzyme inhibition, and antioxidant activities were the assessed indexes in this study (Ayyash, Al‐Dhaheri et al., 2018). Regarding the fact that the water‐soluble extracts of the milk were examined in these studies, the observed health‐beneficial effects are related to the postbiotics present in the extract.

Bacteriocins produced by *Pediococcus pentosaceus* CM16 and *L*. *brevis* CM22 demonstrated strong bacteriocinogenic anti‐listeria activity with important technological properties, for instance, stability over an extensive range of pH (~ 2.0–10.0) and heat resistance (Rahmeh et al., 2019). Some of the probiotic bacteria of raw camel milk have gained interest for their capability to produce EPSs. In a study extracting 82 strains of thermophilic LAB from raw camel milk, selected strains produced EPS in the range 126–319 mg/L for *Streptococcus* strains, 160–740 mg/L for *Lactobacillus* strains, 132–134 mg/L for *Pediococcus* strains, and 70–242 mg/L for *Enterococcus* strains.

EPS produced via *L*. *plantarum* C70 originated from camel milk (with weight‐average molecular weight (Mw) of 3.8 × 10^5^ Da., and glucose (74.6%), arabinose (13.3%), mannose (7.1%), and galactose (5.0%) as its chief monosaccharides) demonstrated cytotoxic actions in contradiction of breast cancer and colon cancer lines. It also has promising properties as an industrial additive due to its shear‐thinning behavior (Ayyash et al., 2020).

**FIGURE 2:**
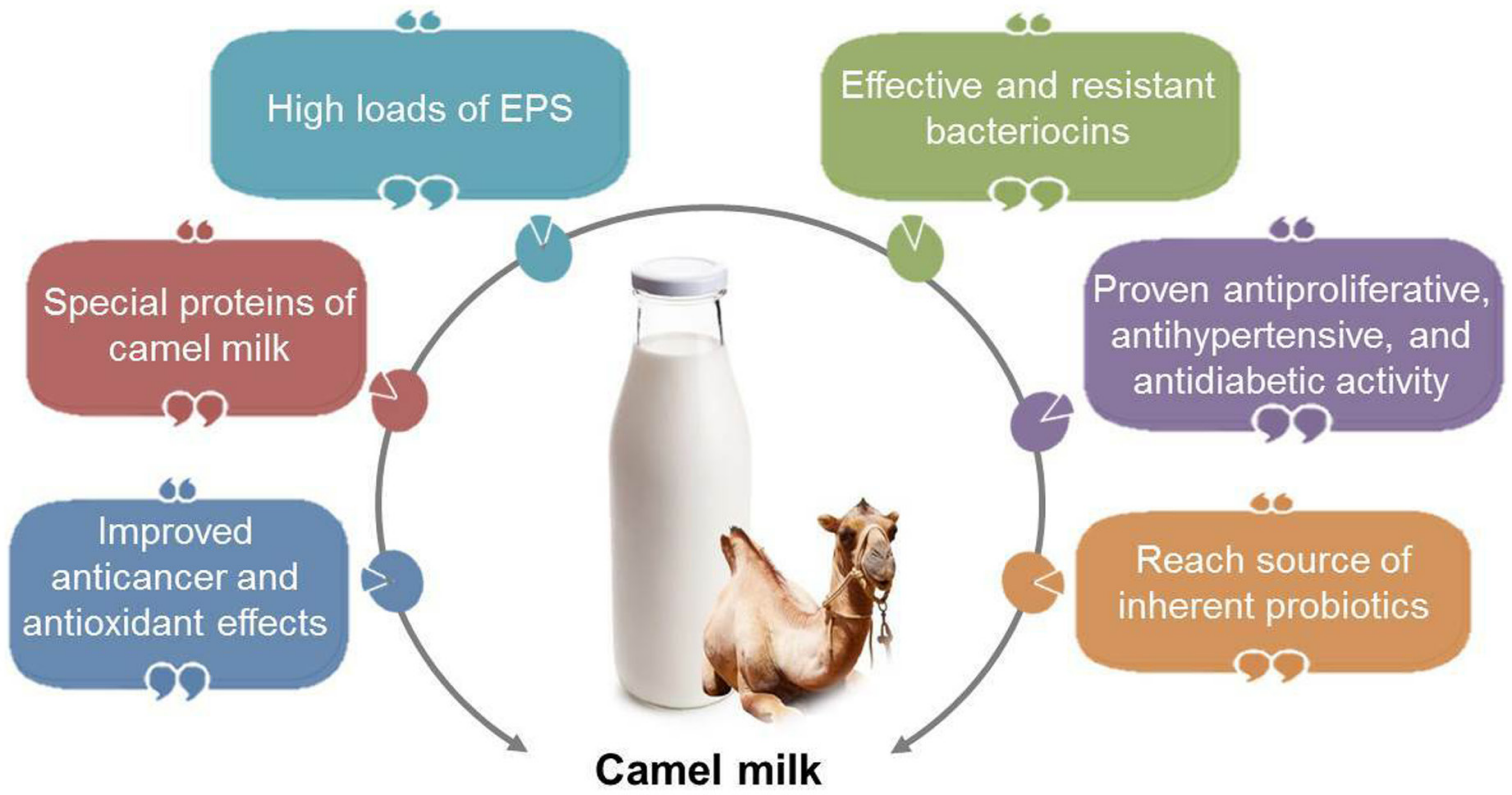
Promising characteristics of camel milk as a carrier for postbiotics.

Fermented camel milk products are rich sources of antioxidant enzymes. For instance, administration of *Lactobacillus paracasei* (obtained from camel milk yogurt) considerably improved the glutathione peroxidase (GSH‐Px) and SOD activities and prevented the malondialdehyde (MDA) as well as protein carbonyl biosynthesis in a dose‐dependent way in the serum kidney, and liver of mice (Wang et al., 2016). The special structure of camel milk can influence the availability and function of the postbiotics. For instance, camel PGRP‐S (as a stable homotetramer) is able to form this property by connecting chains that can interact with lipoteichoic acid, peptidoglycan, and lipopolysaccharide. The protein has originated in non‐mastitis camel milk, nonetheless not in non‐mastitis human or ruminant milk (Hailu et al., 2016). As far as we know, there are no documents exclusively on the postbiotics of camel milk. The above information suggests that it is a promising field for future research (see Figure 2).

## 5 BENEFITS OF PROBIOTICS IN CAMEL MILK

Probiotic camel milk, including *Bifidobacterium lactis*, has been described to keep a lowering of plasma and liver cholesterol concentrations in rats (Ali et al., 2013). In an experimental study, researchers investigated functional outcomes and effects of fermented camel's milk on the lipid profile and liver and kidney action of hypercholesterolemia rats. This study showed that feeding rats with probiotic‐fermented milk ended in a widespread reduction in triglyceride (TG), cholesterol, and LDL in comparison with the positive control group. Albumin and total protein levels increased significantly, but AST, ALT, and creatinine have notably decreased considerably in rats consuming probiotic‐fermented milk. These findings suggest that fermented probiotic milk may enhance liver and kidney capabilities in hypercholesterolemia rats. These results highlighted the potential improvement of camel milk for hyperlipidemia and oxidative stress in rats (Alharbi et al., 2022). In another study, rats‐fed fermented skim camel milk showed lower serum cholesterol levels and LDL‐C/HDL‐C ratio compared to those administered unfermented milk (Yahya et al., 2018).

The mechanism of lowering cholesterol in camel milk is still ambiguous. There are some, such as the interaction between bioactive peptides derived from camel milk proteins and cholesterol, resulting in lower cholesterol levels, as well as the presence of orotic acid in camel milk. It is believed to be responsible for lowering cholesterol levels in humans and rats (Yahya et al., 2018).

Another benefit of camel milk is its antibacterial properties. Due to its high probiotic potential, camel milk can be used to isolate LAB bacteria. Researchers worked on the antibacterial effects of camel milk (Edalati et al., 2019; Mahmoudi et al., 2019). In a study, they isolated LAB bacteria from camel milk and concluded that camel milk has antibacterial properties, especially against *Staphylococcus aureus* and *E*. *coli* bacteria (Edalati et al., 2019).

**FIGURE 3:**
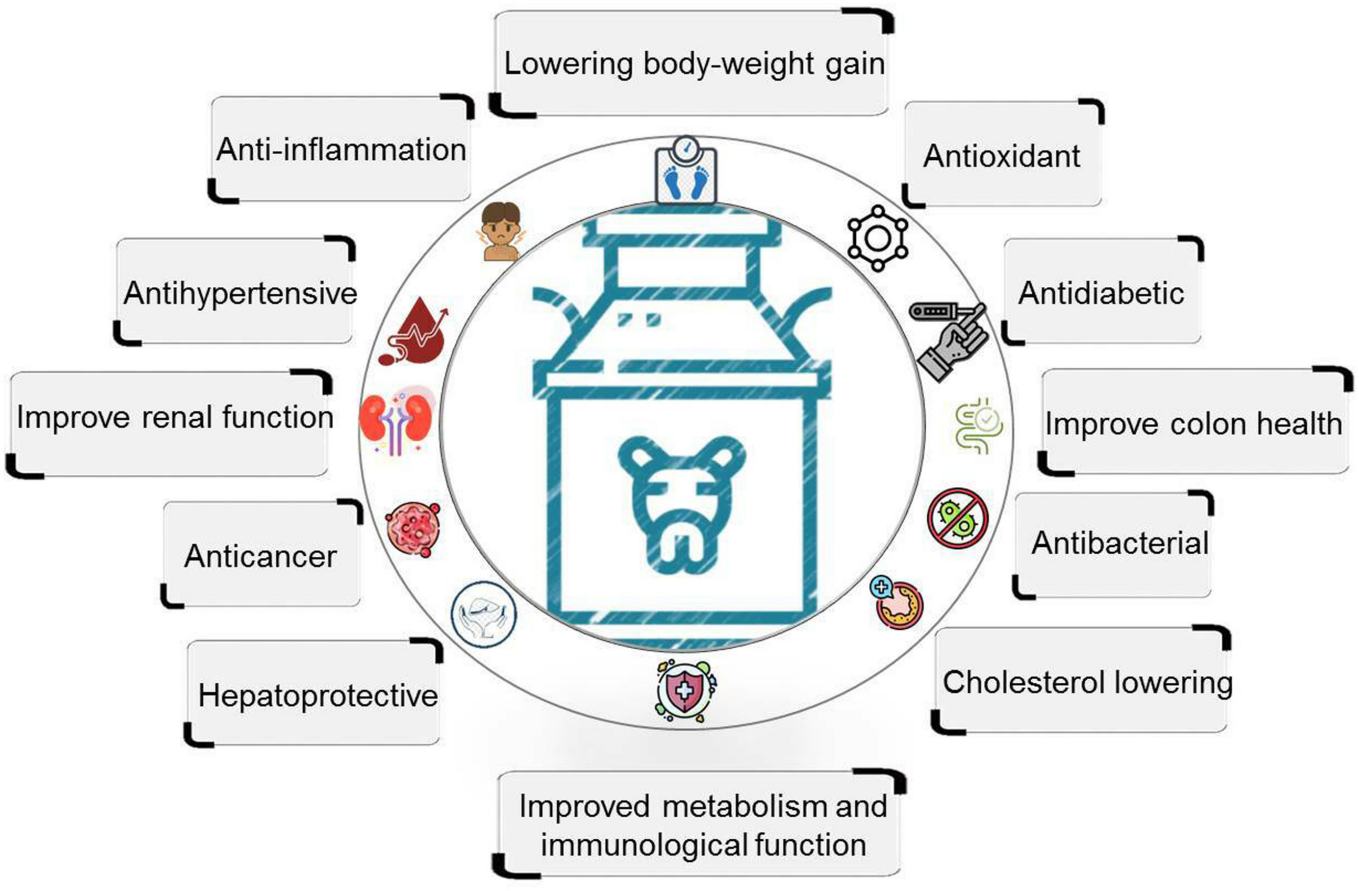
The beneficial effects of the probiotic camel milk and its products.

Recent studies have found that probiotic camel milk has health effects such as improving diabetes and blood pressure, lowering body‐weight gain, cholesterol‐lowering, improving metabolism, immunological function, etc., which can be observed in Figure 3 (Manaer et al., 2021; Shahriari et al., 2018; Swelum et al., 2021). In a recent investigation, researchers have found the positive effects of probiotic camel milk on type 2 diabetes mellitus. In this experimental study of mice, they stated that prepared composite probiotics from camel milk containing *Lactobacillus helveticus*, *Lactobacillus kefiranofaciens*, *Lactococcus lactis*, *Lactobacillus plantarum*, and *Issatchenkia orientalislactobacillus* can decrease fasting blood glucose (FBG), oral glucose tolerance test (OGTT), and glycated hemoglobin (HbAlc) in diabetic mice (Manaer et al., 2021). The antidiabetic effect of camel milk is mainly attributed to existing insulin or insulin‐like growth factors in this milk (Muthukumaran et al., 2022).

Camel milk, unlike the milk of other ruminants, does not form a coagulum in an acidic environment, and this feature causes a low degree of casein phosphorylation to occur in camel milk. So, another possible health benefit of probiotic camel's milk is the improvement of the GI tract. Camel milk is considered a very suitable medium for the delivery of probiotics and can be beneficial in maintaining intestinal health (Vincenzetti et al., 2022).

Some studies have shown the beneficial effects of the probiotic camel milk, which we can observe in Table 3.

**TABLE 3:** Some selected publications on the beneficial effects of the probiotic camel milk and its products.

Product name	Probiotic species	Beneficial effects	References
Fermented camel milk yogurt	*Lactobacillus fermentum*	Decreased microbial load (MRS count) Antioxidant activity (% inhibition of ABTS and DPPH activity) Rancidity formation (TBA value) no foul smell till 7 days of storage	Tak et al. (2018)
Fermented camel milk	ABT‐5^a^	Reduced risk of dyslipidemia associated with metabolic syndrome Lowering body‐weight gain Lowering serum triglycerides and LDL while boosting serum HDL Increased albumin and total protein concentrations Decreased ALT, AST, and creatinine	Alharbi et al. (2022)
Fermented skim camel milk	*Lactobacillus helveticus* (*LMG11445*) *strain + Streptococcus thermophilus* (*ATCC 19258*)	Reduce the risk of hypercholesterolemia Hepatoprotective effects	Yahya et al. (2018)
Fermented camel milk	*Bifidobacterium longum BB536*	Reduced HDL Reduced albumin and total protein Reduced urea and creatinine Reduced ALT and AST Lowering body‐weight gain	El‐Zahar et al. (2021)
Raw camel milk	*Lactococcus lactis* KX881768, *Lactobacillus plantarum* KX881772, *Lactococcus lactis* KX881782, and *Lactobacillus plantarum* KX881779	Auto‐aggregation and high co‐aggregation ability High cholesterol removal ability Strong antimicrobial activity Exopolysaccharides (EPS) production	Abushelaibi et al. (2017)
Low‐fat akawi cheese	*Lactobacillus plantarum*	Angiotensin‐converting enzyme (ACE) inhibition >70% after 7 days of storage‐α‐glucosidase inhibition Increased antiproliferation activity	Al‐Dhaheri et al. (2017)
Fermented camel milk	*Lactococcus lactis* KX881782	α‐glucosidase inhibition Antioxidant activity Angiotensin‐converting enzyme (ACE) inhibition >80% Inhibition of the proliferation of Caco‐2, MCF‐7, and HELA cells	Ayyash, Al‐Dhaheri et al. (2018)

^a^ Contains a mixed strain of *streptococcus thermophilus* TH4, *Lactobacillus acidophilus* LA5, and *Bifidobacterium bifidum* Bb12 at a ratio of 1:1:1.

## 6 ECONOMIC ASPECTS

The production of camel milk is an important economic activity in several regions, including North and East Africa, the Middle East, and parts of Asia. On average, the production of around five liters of milk per day is considered an optimal performance. In Africa, lactating camels typically produce between 1000 and 2700 liters of milk during each lactation period, while their counterparts in South Asia have been known to produce up to 12,000 L. Camels usually reach their peak milk production in the second or third month of lactation, and they continue to produce milk for a period of 8–18 months (Oselu et al., 2022).

Camel milk is much more expensive than regular cow's milk. For example, according to Grand View Research, camel milk sells for about $ 13 per liter in Europe, while cow milk costs about $ 0.40 per liter (GrandViewResearch, 2022). It is estimated that the economic value of camel milk and its by‐product in the global trade is US $ 10 billion per year (Javed, 2019).

In various countries worldwide, camel milk is traditionally drunk in raw or naturally fermented form. Processes such as direct fermentation, pasteurization, cheese, butter, yogurt, powdered milk processing, and production for market have been created in the case of camel milk. Global camel milk production exceeded 8% from 2009 to 2019, indicating significant annual growth (Konuspayeva & Faye, 2021).

Camel milk contains many nutrients, so LAB can quickly produce bio‐functional ingredients that bring health benefits to the consumer. Turkmenistan, Kazakhstan, Mongolia, India, etc., have a wide variety of naturally fermented camel dairy products such as *Gariss*, *Chal*, *Shubat*, *Dhanaan*, *Airag*, *Butsalgaa*, *Arkhi*, *Tsagaa*, *Shmen*, and *Yogurt* (Solanki & Hati, 2018). For example, *Gariss* is special fermented milk, made from camel milk. *Garris* is widely consumed in various parts of Sudan. In addition to stimulating the death of cancer cells, *Garris* consumption may help consumers fight cancer by preventing the formation of new blood vessels that tumors need to grow (Sulieman & Alayan, 2022).

*Chal* is a traditional sparkling beverage of Turkmenistan and in the north of Iran (Golestan province). It is made by fermenting camel's milk at high temperatures for over 4 h. Light, frothy, and refreshing, this drink is famous for its low‐calorie content and high iron, magnesium, calcium, and zinc content. In Kazakhstan, *chal* is known as *shubat* and is usually consumed during the summer (Yam et al., 2014).

*Dhanaan* is common in eastern Ethiopia, mainly in Somalia and the Oromia region, while *Ititu* is made in the Kereyu region of the Oromia (Berhe et al., 2019).

In recent years, the camel milk market has grown rapidly. In 2020, the first adult camel milk powder from the famous brand Aoyou was launched at the Shanghai International Fair. This includes Oz Farm 100% Pure Camel Milk Powder and Oz Farm Probiotic Formula Camel Milk Powder. This is also strong evidence for the development prospects of camel milk. The brand names of some probiotic products derived from camel milk are listed in Table 4.

**TABLE 4:** Some probiotic products derived from camel milk and its economic aspects.

Brand name	Company	Country	Probiotic	Price of product ($)	Weight
Hye food camel milk powder	Aadvik Food and Products Pvt Ltd	India	With probiotic	16.24	500 g
Monch camel milk powder	Monch	India	With probiotic	4.32	50 g
Oz farm camel milk powder	Oz farm	Australia	With probiotic	75	200 g
Camel milk kefir	Desert farms	Saudi Arabi	With probiotic	20	453 g
Camel milk Laban	Camelicious	Dubai	With probiotic	1.36	250 mL

The global probiotic market trend is stated on a markets and markets website. This website noted that the probiotics market is projected to grow from USD 65.9 billion in 2022 to 91.1 billion by 2026. The researchers on this website estimated the sales of probiotic camel milk to be $3000 this year worldwide (MarketsandMarkets, 2022).

Several studies have examined the economic aspects of probiotic camel milk (Gebremichael et al., 2019; Konuspayeva et al., 2022). These studies concluded that the high costs associated with the production of camel milk in concentrated agricultural establishments are significant, and the financial outcome of a concentrated camel farm can only be profitable if the selling price of milk is significantly increased. Currently, in the majority of nations engaged in production, camel milk is sold at a value that is more expensive than the value of bovine milk (Konuspayeva et al., 2022). However, the benefits of probiotic camel milk, such as easy digestibility, more amount of vitamin C, a high iron content, polyunsaturated fatty acids, anti‐inflammatory proteins, the presence of lactoferrin, immunoglobulins, and the presence of insulin, have made its consumption preferable to cow's milk (Darani et al., 2023).

The processing of camel milk faced several challenges, such as the limited stability of the milk when subjected to UHT treatment, the creation of fragile curd during coagulation, decreased ability of rennet, extended fermentation period, and reduced thermal stability during the drying process. Therefore, the production of cheese from camel milk takes a longer time to coagulate. Also, for yogurt production, these features cause the produced yogurt to be less firm and fragile (Seifu, 2023). However, the production and supply of probiotic camel milk is cost‐effective due to the presence of LABs that facilitate the fermentation process (Sheikh et al., 2024).

## 7 CHALLENGES AND PERSPECTIVES

For a considerable period, camel milk was solely enjoyed by the herdsmen and held no commercial value. Furthermore, apart from undergoing fermentation for preservation purposes, no alterations were made to the camel milk (Konuspayeva & Faye, 2021). Supplying camel milk to the national or international market is one of the goals that is being pursued recently (Faye, 2016). To commercialize and expand the production of camel milk, it is important to develop national/regional/international standards for it, and considering the differences between camel milk and cow's milk, cow's milk standards should not be used in camel milk production. Also, for the pasteurization of camel milk, the relevant conditions and indicators must be determined. Actually, a preliminary study showed that alkaline phosphatase (ALP), which is used for cow milk (Rankin et al., 2010), was not a suitable indicator of pasteurization of camel milk, because camel ALP is resistant to heat and is still active at 90°C (Elagamy, 2000). Lorenzen et al. (2011) stated that lactoperoxidase (LPO) can be a suitable indicator of pasteurization. Tayefi‐Nasrabadi et al. (2011) confirmed that camel milk LPO has lower heat resistance than cow milk LPO. Such doubts about choosing an appropriate index for the pasteurization of camel milk create limitations to achieving an international standard. One of the effective factors in the commercialization of camel milk is its sensory properties after thermal processes. Few studies have been done on the sensory characteristics of camel milk after heat treatment. In one of these studies, Lund et al. (2020) compared the effects of three different heat treatments and observed that the treated samples showed lower taste, texture, and overall acceptance scores than the control samples. The use of starter cultures that contain a limited number of live microbial strains (single or mixed), and their inoculation in raw camel milk leading to controlled fermentation, can lead to the expected sensory properties according to consumers' tastes in the final product. However, previously fermented milk is usually used to inoculate raw milk, in which case the fermentation process is spontaneous and, as a result, a complex microflora ecosystem is created in fermented milk, which causes the creation of final products with highly variable characteristics, that among them, it is difficult to find products with standard sensory characteristics. For example, Shubat is a product that is produced by spontaneous fermentation, which usually results in gas and foam production, and sometimes becomes a particularly acidic product that urban consumers are reluctant to use (Berzhanova et al., 2014). Although some starters were commercialized industrially recently, considering the many strains present in naturally fermented milk, additional studies on the technological characteristics of these strains are needed (Ibrahim et al., 2009). In addition to the development of fermented camel milk with desirable and standardized sensory properties, the use of a suitable probiotic bacterium also provides potential health benefits. For example, the inoculation of camel milk with *Lactococcus lactis* KX881782 isolated from raw camel milk showed therapeutic properties including inhibition of alpha‐glucosidase (antidiabetic effect), antioxidant activity, inhibition of the angiotensin‐converting enzyme (antihypertensive effect), and antiproliferative activity (anticancer effect) than cow milk (Ayyash, Al‐Dhaheri et al., 2018). The consumption of probiotic‐fermented camel milk, as well as its probiotic quality, which confers the health effects of the product, is an important commercial debate (Manaer et al., 2015). It is a necessary issue that probiotics have the ability to ferment milk as well as survive during the storage of fermented milk to provide health benefits to the host. In past studies, it has been determined that the probiotics in camel milk have a long shelf life, and after using these probiotics to ferment the milk, their viability is maintained at an optimal level to provide health benefits (Abushelaibi et al., 2017; Angmo et al., 2016; Didar, 2019). In addition, raw camel milk and its fermented products can be a good source of potential probiotic strains, because a mixture of different bacterial species and yeasts have been identified in camel milk (Ider et al., 2019; Shori, 2017). Of course, to produce a variety of probiotic products and develop them, the strains must be selected based on functional criteria, because the selection of inappropriate probiotic strains can lead to the production of undesirable products (Tropea, 2015). As an example of a suitable choice, fermentation of camel milk using *Lactobacillus fermentum*, *Lactobacillus rhamnosus*, and *Lactobacillus plantarum* PTCC 1058 leads to desirable sensory properties and also the production of a high amount of bioactive peptides in milk (Moslehishad et al., 2013; Nanda et al., 2011).

In general, camel milk has potential health benefits for several diseases such as diabetes, allergic conditions, and cancer, and “probiotic camel milk” has been introduced as a beneficial product with advanced health properties. These characteristics are associated with the function of the probiotic bacteria and the effects of the produced postbiotics. Regarding the above facts, it is an attractive field of investment for a growing market. It is especially considering that camel is resistant to harsh environmental conditions and lack of water; therefore, camel milk is considered an important dairy product, especially in countries that encounter increased damage to agricultural and water resources.

## 8 CONCLUSIONS

Camel milk has shown many advantages over milk from other animals and presents promising opportunities for health and economic sectors due to its unique properties and potential benefits. Probiotic isolates from camel milk, such as LAB and Bifidobacteria, show potential as probiotics with beneficial properties. The predominance of β‐casein over other casein parts, and the deficiency of β‐lactoglobulin, which cause low allergenicity of milk, along with high nutritional value, make camel milk consumption suitable for humans, especially children who are sensitive to cow's milk. Additionally, postbiotics derived from camel milk exhibit antioxidant, antidiabetic, antiproliferative, and antihypertensive effects. The economic aspects of camel milk production were also explored, noting its increasing global demand and potential for commercialization. Despite the challenges in standardizing processing methods and ensuring sensory quality, the potential health benefits, economic opportunities, and versatility of camel milk and its probiotic derivatives make it a promising field for further research and development.

## AUTHOR CONTRIBUTIONS

**Fereshteh Ansari:** Conceptualization (equal); writing – original draft (equal). **Hadi Pourjafar:** Conceptualization (equal); investigation (lead); supervision (lead); writing – original draft (equal); writing – review and editing (equal). **Shohreh Alian Samakkhah:** Writing – original draft (equal); writing – review and editing (equal). **Esmaeel Mirzakhani:** Writing – original draft (equal); writing – review and editing (equal).

## CONFLICT OF INTEREST STATEMENT

The authors declare that they do not have any conflict of interest.

## ETHICS STATEMENT

This study does not involve any human or animal testing.

## Data Availability

Not applicable.
